# Unusual Histopathological Findings in Mechanically Removed Stroke Thrombi – A Multicenter Experience

**DOI:** 10.3389/fneur.2022.846293

**Published:** 2022-05-17

**Authors:** Oskar Aspegren, Senna Staessens, Sarah Vandelanotte, Linda Desender, Charlotte Cordonnier, Laurent Puy, Nicolas Bricout, Simon F. De Meyer, Tommy Andersson, Fabian Arnberg

**Affiliations:** ^1^Department of Pathology and Cancer Diagnostics, Karolinska University Hospital, Stockholm, Sweden; ^2^Department of Oncology-Pathology, Karolinska Institutet, Stockholm, Sweden; ^3^Laboratory for Thrombosis Research, KU Leuven Campus Kulak Kortrijk, Kortrijk, Belgium; ^4^University of Lille, INSERM, CHU Lille, U1172-Lille Neuroscience & Cognition (LilNCog), Lille, France; ^5^Department of Interventional Neuroradiology, CHU Lille, Lille, France; ^6^Department of Medical Imaging, AZ Groeninge, Kortrijk, Belgium; ^7^Department of Clinical Neuroscience, Karolinska Institutet, Stockholm, Sweden; ^8^Department of Neuroradiology, Karolinska University Hospital, Stockholm, Sweden

**Keywords:** ischemic stroke, mechanical thrombectomy, calcification, extracellular DNA, myofibroblast, adipocyte-like, collagen, myxomatous

## Abstract

**Background:**

Several studies have investigated the histopathology of mechanically retrieved thrombi from stroke patients. Thrombi with unusual components constitute about 1–2% of all stroke thrombi in clinical practice. Knowledge about these rare components is limited.

**Objectives:**

To characterize the histopathology of unusual stroke thrombi from a real-world setting with relation to clinical presentation, patient characteristics and procedural aspects of mechanical thrombectomy.

**Methods:**

One-thousand and eight thrombi retrieved from stroke patients with mechanical thrombectomy at three different hospitals were retrospectively reviewed for unusual histological components. Fifteen thrombi were included in the study for further histopathological analysis. Clinical data and data on procedural aspects were collected.

**Results:**

We identified six cases with large amounts of extracellular DNA, of which three were calcified. All six cases except one received anticoagulant therapy. We describe two types of calcifications that differ with respect to general calcification morphology, von Kossa staining pattern, macrophage immunophenotype and presence of multinucleated giant cells. Cholesterol-rich (*n* = 3), adipocyte-like pattern-rich (*n* = 2), collagen-rich (*n* = 2) and myxomatous (*n* = 1) thrombi were also identified and are discussed with regard to pathogenesis and clinical and intervention characteristics. Finally, a thrombus with parts of a vascular wall is described. Suggestions for future studies are made and clinical and technical aspects of the management for these rare but important patients are discussed.

**Conclusion:**

In our retrospective multicenter study, we characterized stroke thrombi histopathologically and found subgroups of thrombi defined by presence of rarely seen components. These defined subgroups showed relation to underlying cardiovascular disease, patient characteristics, and mechanical thrombectomy technique. Knowledge about these components may increase our understanding of stroke pathophysiology and influence interventional procedures.

## Introduction

Since the pivotal studies published in 2015 (1–5), mechanical thrombectomy (MT) has been established as standard treatment for patients with acute ischemic stroke (AIS) due to large vessel occlusion. As retrieved stroke thrombi have become available for systematic histopathological examination, a new field within stroke pathology has emerged. Standardized protocols for basic histological processing of stroke thrombi (6) provide consistent analysis of thrombi and identify common and unusual morphological variations. About 98% of all removed stroke thrombi can be defined as having varying amounts of four elemental components, i.e.,: (I) red blood cells (RBCs), (II) fibrin, (III) platelets, and (IV) leukocytes, all deriving from human whole blood. In addition, von Willebrand Factor (vWF) and neutrophil extracellular traps are found in varying amounts in the majority of stroke thrombi. The remaining 1–2% of stroke thrombi contain unusual and unexpected elements such as mineralization (7–11), large amounts of extracellular DNA (ecDNA) (12, 13), cholesterol, adipocyte-like patterns (8), bacterial vegetations (9), collagen and vascular wall components (14). Whether presence of such unusual elements is associated to certain health disorders (e.g., large artery atherosclerosis), impacts technical aspects of MT or affects clinical outcome, is largely unknown. It is also unclear why and under which circumstances these unusual and unexpected elements appear in stroke thrombi.

The intention of this descriptive study is to retrospectively define subgroups of stroke thrombi by presence of rare histopathological elements by a synthesis of interesting observations from three European university hospital centers with several years of experience regarding histopathological analysis of stroke thrombi. We aim to contribute to the understanding of thrombus composition with special reference to unusual elements detected by histopathological examination. In addition, we would like to stimulate further studies on unusual thrombus components in relation to possible radiological recognition, influence on the thrombectomy procedure and potential origin.

## Materials and Methods

### Collection of Patient Thrombi

Thrombi collected from acute ischemic stroke patients undergoing mechanical thrombectomy at Karolinska University Hospital (KUH) in Stockholm, Sweden, AZ Groeninge Hospital (AZGH), Belgium and Lille University Hospital (LUH), France, were retrospectively reviewed and searched for unusual components. The inclusion criteria were stroke patients above the age of 18 years, treated with MT for a large vessel occlusion with thrombus material available for histopathological analysis, irrespective of the date of mechanical thrombectomy and histopathological analysis. That is, all stroke thrombi available at each of three centers were reviewed. All thrombi were retrieved using a stent retriever and/or aspiration device. At KUH, all stroke thrombi between February 2019 to November 2021 were retrospectively identified in the laboratory information system and reviewed (*n* = 507). The same procedure was carried out at AZGH and LUH where 267 and 234 specimens were identified from the period October 2014 until December 2019 and August 2017 until December 2019, respectively. In total, 1008 thrombi were reviewed, of which fifteen were included as thrombi with unusual features. Eight of these cases were identified at KUH, four at LUH and three at AZGH. From the onset of the COVID-19 pandemic in February 2020 to November 2021, 373 patients were thrombectomized at KUH. Thrombi were selected and included based on histopathological features only, without regard of any clinical and/or radiological characteristics.

### Clot Processing and Histopathological Analysis

At all three medical centers, thrombi were removed from the device and immediately incubated in 10% phosphate buffered formalin for at least 24 h at room temperature. The histopathological protocol was carried out in accordance with internationally established methods (6). Thrombi collected from AZGH and LUH were processed at the Laboratory for Thrombosis Research at the Catholic University of Leuven Campus Kortrijk (Kulak), Belgium. Irrespective of medical center, whole samples were embedded in paraffin and at least three consecutive 5 μm sections of the thrombus were cut and stained with Hematoxylin & Eosin (H&E) for general morphology, Martius Scarlet Blue (MSB) for fibrin, and immunohistochemically with anti-CD42b (GPIbα, MA5-11642, Invitrogen, Waltham, MA, USA) for identification of platelets. None of the sections were decalcified prior to staining. At Kulak, all thrombi were additionally routinely stained with Feulgen's reaction [DNA staining, 1079070001, Merck Chemicals, MA, USA] and immunohistochemically stained with the antibodies anti-vWF for identification of von Willebrand factor (A008202-2, Dako, Glostrup, Denmark), anti-CD45 for leukocytes (Receptor-type Tyrosine-protein Phosphatase C, 304002, Biolegend, San Diego, CA, USA) and anti-H3Cit for neutrophil extracellular traps (Citrullinated histone H3, ab5103, Abcam, Cambridge, UK). At KUH, the following antibodies were used when deemed appropriate: anti-α-SMA for myofibroblast differentiation (Alpha-Smooth Muscle Actin, 1A4, Ventana), anti-AE1/AE3 for keratin expression (Pan Keratin, AE1/AE3/PCK26, Ventana), anti-Calretinin for identification of myxoma cells and adipocytes (Calretinin, SP65, Ventana), anti-CD31 for endothelial cells (CD31/PECAM-1, JC70, Ventana), anti-CD34 for endothelial cells (CD34, QBEND/10, Ventana), anti-CD42b for platelets (GP1BA, 42C01, ThermoFisher Scientific), anti-CD45 for leukocytes (CD45/Receptor-type Tyrosine-protein Phosphatase C, RP2/18, Ventana), anti-CD68KP1 for myeloid precursors and macrophages (CD68, KP1, Ventana), anti-CD68PG-M1 for macrophages (CD68, PG-M1, Ventana), anti-CKMNF for keratin expression (Pan Cytokeratin, MNF 116, Dako), anti-FVIII for Factor VIII (Coagulation factor VIII, Ventana), anti-GrB for Granzyme B (Granzyme B, GrB-7, Monosan), anti-Ki67 for cell proliferation (Ki-67 Rabbit, 30-9, Ventana), anti-MPO for neutrophils (Myeloperoxidase, Ventana), anti-NSE for myxoma cells (Neuron-Specific Enolase, MRQ-55, Ventana), anti-PGP9.5 for myxoma cells (Protein Gene Product 9.5, 31A3, BioRad), anti-S100 for adipocytes (S100, 4C4.9, Ventana), anti-SATB2 for osteoblasts (SATB2, EP281, Cell Marque), and anti-Vimentin for myxoma cells (Vimentin, V9, Ventana). In all immunohistochemical stains at KUH, DAB (3,3′-Diaminobenzidine) was used as chromogen and hematoxylin as counterstain. Special histochemical stains used at KUH were, in addition to MSB, von Kossa to confirm presence of calcium, Feulgen's reaction to confirm presence of DNA, Periodic Acid Shiff (PAS) for glycogen, neutral polysaccharides and glycoproteins, Periodic Acid-Schiff with diastase (PAS-D), for comparison with PAS with glycogen removed, Grocott's methenamine silver stain for fungi, Gram-stains to identify bacteria, Giemsa for microorganisms, Elastic van Gieson (Verhoeff van Gieson) stain to confirm elastic fibers and finally Perls (Prussian Blue) stain for identification of iron.

The histological examination of the specimens from KUH was performed by OA and cases from Kulak were examined by OA, SS and SV. All included cases were reviewed using both light microscopy and digitized slides, utilizing whole slide scanning with Hamamatsu NanoZoomer Digital slide scanner (at x40, with model S360 at KUH and at x20, with model SQ at Kulak) (Hamamatsu Photonics, Japan).

### Clinical and Radiological Data

Technical details of concern to the thrombectomy procedure were collected, including occluding thrombus location, number of passes, modified Thrombolysis in Cerebral Infarction (mTICI) score and procedural time. In addition, data on dense vessel sign were collected at KUH and data on specific devices were collected and AZGH/LUH, respectively. The clinical data collected from all three centers were age, gender, medical history [prior stroke/transient ischemic attack (TIA), atrial fibrillation, hypertension, diabetes, and tobacco abuse], anticoagulant use, National Institutes of Health Stroke Scale (NIHSS) score at admission, use of recombinant tissue plasminogen activator (rtPA), NIHSS at 24 h post-MT and the functional outcome at 90 days post MT assessed by modified Rankin Scale (mRS) (15). Stroke etiology was self-reported at each center and classified using the Trial of Org 10172 in Acute Stroke Treatment (TOAST) system (i.e., (I) Large Artery Atherosclerosis (LAA), (II) cardioembolic (CE), (III) stroke of other determined etiology and (IV) cryptogenic (16). In addition, data on pre-MT mRS and platelet aggregation inhibitor medication status were available at AZGH and LUH, while history of heart failure and specific type of anticoagulant were available at KUH.

At KUH, radiological and interventional data were obtained from the Swedish Register for Endovascular Treatment of Stroke (EVAS) and all clinical data was obtained from the Swedish National Stroke Register (Riksstroke). At AZGH, clinical and interventional data were obtained from the local Register for Endovascular Treatment of Stroke (EVAS-BE), whereas the data were collected from the in-hospital stroke register at LUH.

This study was conducted in accordance with the ethical standards of the Declaration of Helsinki and its amendments. Furthermore, it was approved by the Swedish Research Ethics Board (2020-03349). All patients and/or their legal representative were informed under the approval of the AZGH ethical committee (AZGS2015065) or the French ethical committee (2019-A00414-53) at LUH.

## Case Presentations

In our data set of 1008 patient thrombi in total, we identified fifteen thrombi from 15 individuals that contained seven unusual and unexpected features. These thrombi contained significant amounts of (1) ecDNA with calcifications, (2) ecDNA without calcifications, (3) Cholesterol, (4) Adipocyte-like structures, (5) Connective tissue, (6) Myxomatous material, and (7) Vascular wall components.

Clinical and intervention characteristics are presented in Tables 1–3.

**Table 1:** Clinical characteristics.

**Case**	**Category**	**Age**	**G**	**Prior stroke/TIA**	**HF**	**AF**	**HT**	**DM**	**Smoking**	**AC**	**AA**
1.1	Microcalcific ecDNA	77	F	No	No	Yes	Yes	No	No	Yes	No
1.2	Large calcific ecDNA	78	F	Yes	Yes	Yes	Yes	Yes	No	Yes	No
1.3	Large calcific ecDNA	85	F	Yes	NA	Yes	No	No	No	No	Yes
1.4	ecDNA-rich	81	F	No	NA	Yes	Yes	Yes	No	Yes	No
1.5	ecDNA-rich	75	F	No	NA	Yes	Yes	Yes	No	Yes	No
1.6	ecDNA-rich	78	F	No	NA	Yes	Yes	Yes	No	Yes	No
2.1	Cholesterol necrosis	76	M	Yes	No	Yes	Yes	No	No	No	No
2.2	Cholesterol necrosis	73	M	Yes	NA	No	Yes	Yes	Yes	No	Yes
2.3	Cholesterol inflammation	75	M	No	No	No	Yes	No	No	No	No
3.1	ALP	53	M	Yes	No	No	No	No	No	No	Yes
3.2	ALP	72	F	Yes	NA	Yes	No	Yes	No	No	Yes
4.1	Collagen-rich	73	M	No	No	No	Yes	No	No	No	No
4.2	Collagen-rich	52	M	No	NA	No	No	No	Yes	No	No
5	Myxomatous material	79	F	No	No	No	Yes	Yes	No	No	Yes
6	Vascular wall	52	F	No	No	No	No	No	No	No	No

*AA, Antithrombotic agent; AC, Anticoagulation; AF, Atrial fibrillation; ALP, Adipocyte-like pattern; DM, Diabetes mellitus; ecDNA: extracellular deoxyribonucleic acid; F, Female; G, Gender; HF, Heart failure; HT, Hypertension; M, Male; TIA, Transient ischemic attack*.

**Table 2:** Neurologic disability of included patients and stroke etiology according to TOAST-system.

**Case**	**Category**	**NIHSS arrival**	**NIHSS 24 h**	**NIHSS diff**	**mRS preStroke**	**mRS 90 days**	**TOAST**
1.1	Microcalcific/ecDNA	14	0	−14	NA	1	CE
1.2	Large calcific/ecDNA	24	19	−5	NA	6	CE
1.3	Large calcific/ecDNA	20	2	−18	NA	3	LAA
1.4	ecDNA-rich	19	9	−10	1	NA	LAA
1.5	ecDNA-rich	20	13	−7	2	4	CE
1.6	ecDNA-rich	24	6	−18	NA	3	CE
2.1	Cholesterol, necrosis	21	22	1	NA	6	LAA
2.2	Cholesterol, necrosis	23	20	−3	2	NA	Other
2.3	Cholesterol, inflammation	NA (GCS 3)	NA (GCS 3)	NA	NA	6	Crypt.
3.1	Adipocyte-like pattern	5	11	6	NA	1	Crypt.
3.2	Adipocyte-like pattern	19	3	−16	NA	4	Crypt.
4.1	Collagen-rich	16	0	−16	0	0	Crypt.
4.2	Collagen-rich	22	1	−21	NA	1	Crypt.
5	Myxomatous material	11	23	12	NA	6	Crypt.
6	Vascular wall	8	3	−5	NA	0	Crypt.

*ecDNA, extracellular deoxyribonucleic acid; CA, Cardioembolic; Crypt., Cryptogenic; diff: difference; GCS,Glasgow coma score; LAA, Large artery atherosclerosis; mRS: modified Rankin Score; NA, Not applicable; NIHSS, National Institutes of Health Stroke Scale; TOAST, Trial of Org 10,172 in Acute Stroke Treatment*.

**Table 3:** Intervention characteristics.

**Case**	**Category**	**Occl. vessel**	**Dense vessel sign**	**rtPA**	**mTICI**	**Nr. of passes**	**Proc. time (mins)**	**Device**
1.1	Microcalcific ecDNA	M1	No	No	3	2	46	NA
1.2	Large calcific ecDNA	M1	No	No	2b	7	119	NA
1.3	Large calcific ecDNA	MCA	NA	Yes	3	1	12	Aspiration
1.4	ecDNA-rich	MCA	NA	No	3	1	35	Embotrap
1.5	ecDNA-rich	ICA-T	NA	No	2a	1	27	Embotrap
1.6	ecDNA-rich	ICA-T	NA	No	3	2	20	Stent solitaire + aspiration
2.1	Cholesterol necrosis	ICA T	Yes	No	0	2	210	NA
2.2	Cholesterol necrosis	MCA	NA	No	3	1	25	Embotrap
2.3	Cholesterol inflammation	BA	No	No	2a	7	110	NA
3.1	ALP	ICA T	No	Yes	3	4	108	NA
3.2	ALP	ICA	NA	No	3	3	23	Aspiration + stent Trevo
4.1	Collagen-rich	M1	Yes	Yes	3	1	8	Embotrap
4.2	Collagen-rich	BA	NA	Yes	1	3	31	Stent Trevo + aspi. Sofia
5	Myxomatous material	M2	Yes	No	2b	5	190	NA
6	Vascular wall	M2	Yes	No	2c	1	40	NA

*ALP, Adipocyte-like pattern; BA, Basilar artery; ecDNA, extracellular deoxyribonucleic acid; ICA-T, Internal carotid artery, T-occlusion; M1, First segment of the middle cerebral artery; M2, Second segment of the middle cerebral artery; MCA, Middle cerebral artery; mTICI, Modified treatment in cerebral ischemia score; NA, Not available; Occl. vessel, Occluded vessel; Proc, Procedure time, i.e., groin puncture to end of procedure; rtPA, Recombinant tissue plasminogen activator*.

### Extracellular DNA With/Without Calcifications

#### Case 1.1–6

##### Clinical Data

We identified six cases of stroke thrombi with considerable amounts of ecDNA, of which three were mineralized. All six patients were elderly females with an average age of 79 years. Cardiovascular risk factors were frequent among these patients and two of them had a history of stroke/TIA. All women, except one, had a history of atrial fibrillation and were treated with either non-vitamin K antagonist oral anticoagulants (NOAC) or warfarin. The patient with no known atrial fibrillation received treatment with oral anti-platelet medication. The pre-stroke mRS was on average assessed to 1, 5. At admission, all six patients had an occluded ICA-T or MCA and the average NIHSS score was 20 (median 20). All underwent MT without prior rtPA, except for the case with anti-platelet aggregation. MT outcomes were considered successful in this group with a median mTICI score of three and an average number of 2, 3 passes (median 1, 5) with an average procedure time of 38 min (median 31 min). 24 h post MT, the average NIHSS-score was 8,2 (median 7, 5) and the mRS score at 3 months was 3, 4 (median 3). Stroke etiology was clinically assessed as cardioembolic in four cases and large artery atherosclerosis in two. One of the patients died of SARS-CoV-2 disease 3 weeks post-MT.

##### Histological Examination

Common to these six thrombi were webs of basophilic material interpreted as ecDNA based on positive Feulgen stains. They were large enough to create complex trabecular patterns. Besides the absence of neutrophils morphologically, there was lack of H3Cit-immunoreactivity within the ecDNA in four out of six cases, indicating a non-neutrophil source of the ecDNA.

#### Case 1.1–1.3

**Figure 1:**
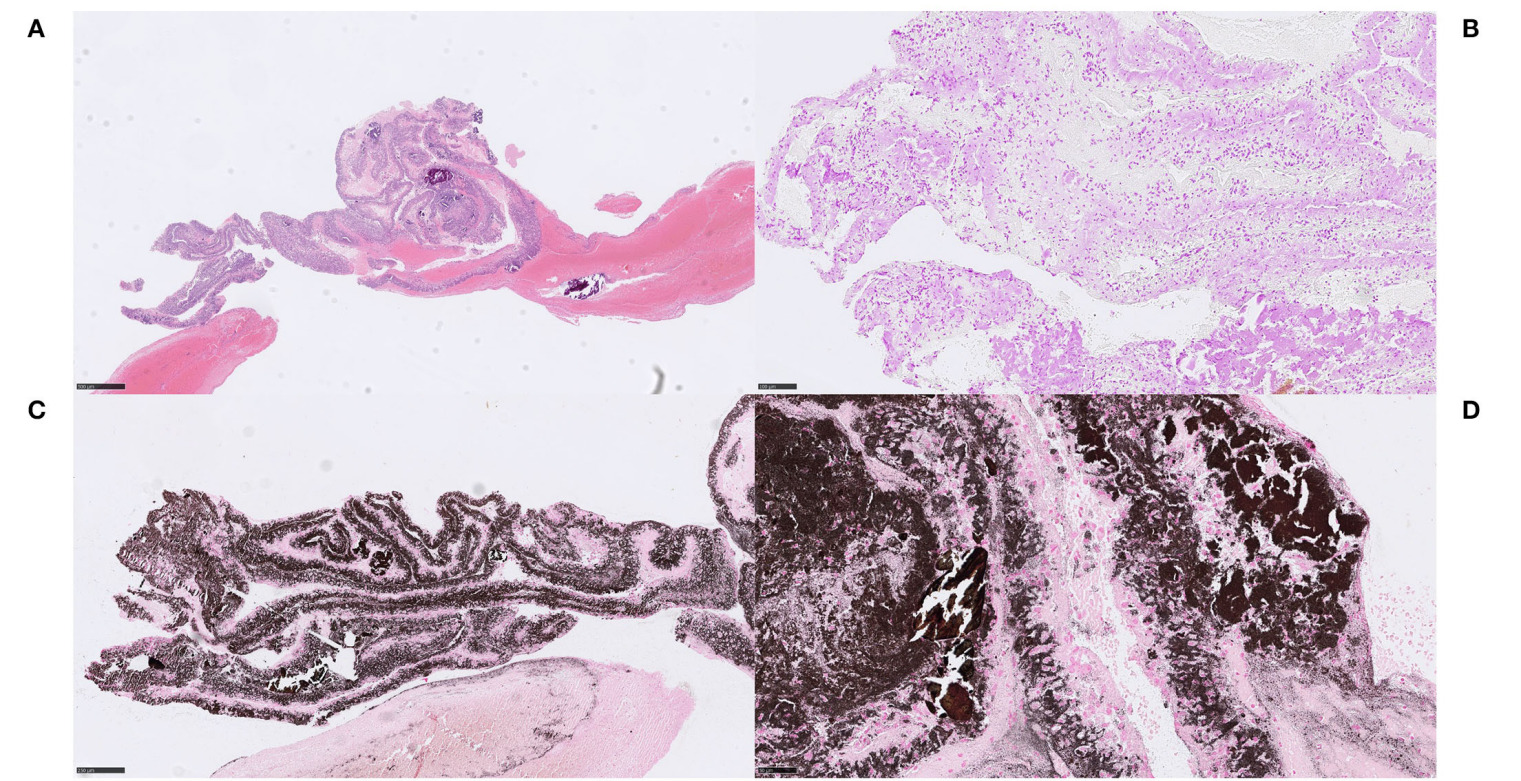
Extracellular DNA (ecDNA) with calcifications. Large calcific type. Case 1.2: Large calcific type displayed large calcific crystals scattered within the thrombus along with webs of basophilic material **(A)** H&E, 50x. The basophilic material was stained with DNA stain (Feulgen), confirming its DNA content and was thus considered to be ecDNA **(B)** Feulgen, 100x. In addition, the ecDNA was found to be lightly mineralized by calcium, visualized with the calcium stain von Kossa **(C)** von Kossa, 100x. On high power, pieces of partly broken calcific crystals, showing dense and homogenous von Kossa staining pattern, were seen in conjunction with the ecDNA, showing relatively thin and grainy staining pattern **(D)**, von Kossa, 400x. Consequently, ecDNA in stroke thrombi coexist with large plaques of calcifications and the ecDNA itself contains at least calcium in addition to nucleic acids. Formation of large calcific plaques is most likely a result of pre-symptomatic processes within atherosclerotic plaque or aortic valves. Calcific plaques exposed to blood flow may be followed by colonization of circulating monocytes and an on-site rapid release of DNA as monocyte extracellular traps, possibly associated with the release of calcium. Plaque rupture in a large artery or a heart valve may therefore be the source of these emboli. *For 50x, scale bar* = *500* μ*m; for 100x, scale bar* = *250* μ*m; for 400x, scale bar* = *50* μ*m*.

**Figure 2:**
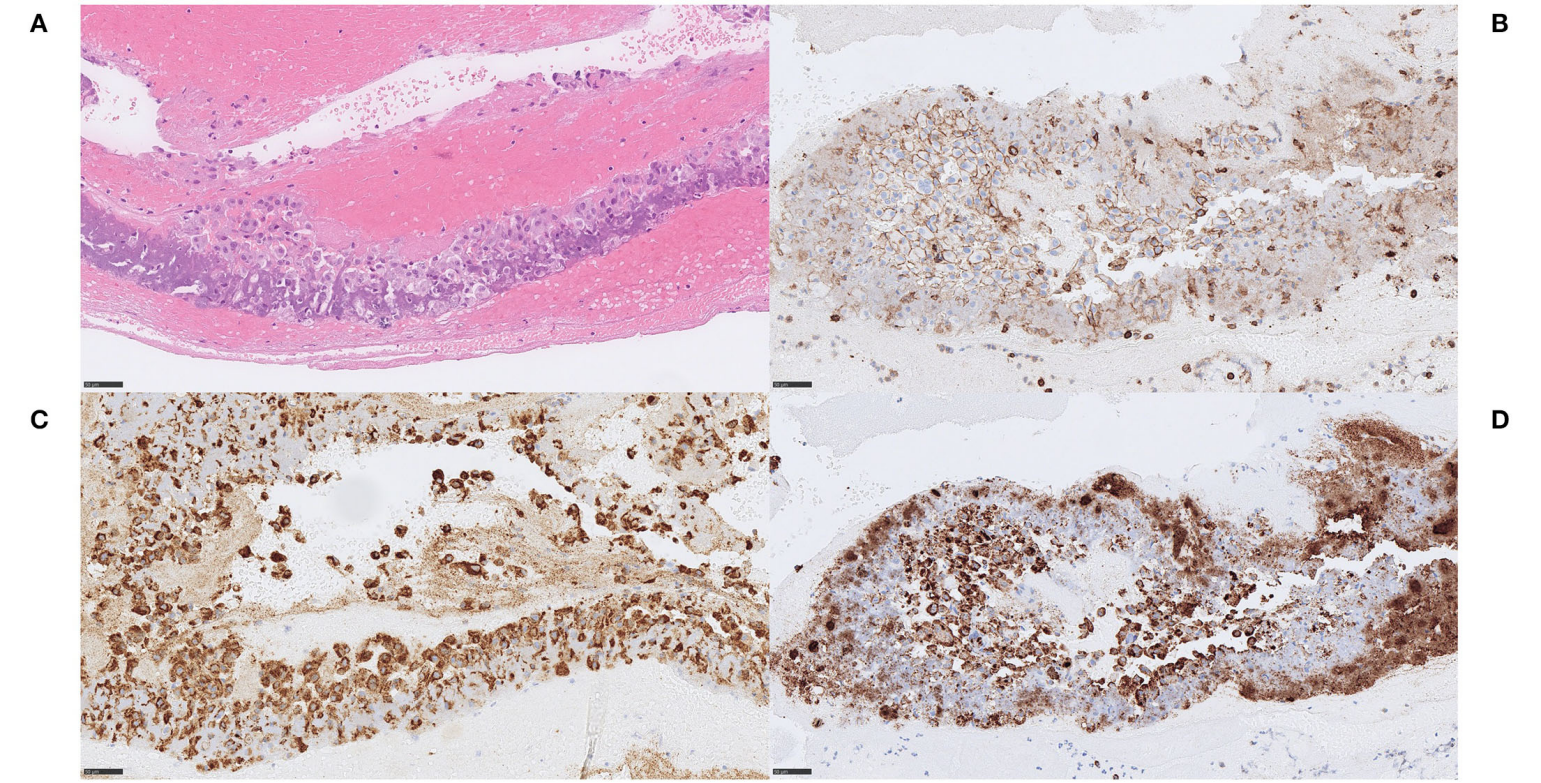
Extracellular DNA (ecDNA) with calcifications. Large calcific type. Case 1.2: The cell population that was identified in relation to ecDNA and large calcific plaques showed various morphology of both moderately large cells with abundant cytoplasm, rounded and eccentrically placed nuclei with pale, evenly distributed chromatin, suggestive of macrophages **(A)** H&E, 400x, as well as spindle-shaped cells with long, very eosinophilic cytoplasmic extensions reminiscent of myofibroblats (not shown). However, all cells showed the same immunophenotype of strong immunoreactivity for the pan-leukocytic marker CD45 **(B)** CD45, 400x, the macrophage marker CD68 **(C)** CD68PG, 400x and the smooth muscle cell marker α-SMA **(D)**, α-SMA, 400x, indicative of a macrophage-to-myofibroblast transition. Considering the amounts of ecDNA and the type of cells present in these thrombi, the ecDNA may represent a type of monocyte-extracellular traps (METs). S*cale bar* = *50* μ*m*.

**Figure 3:**
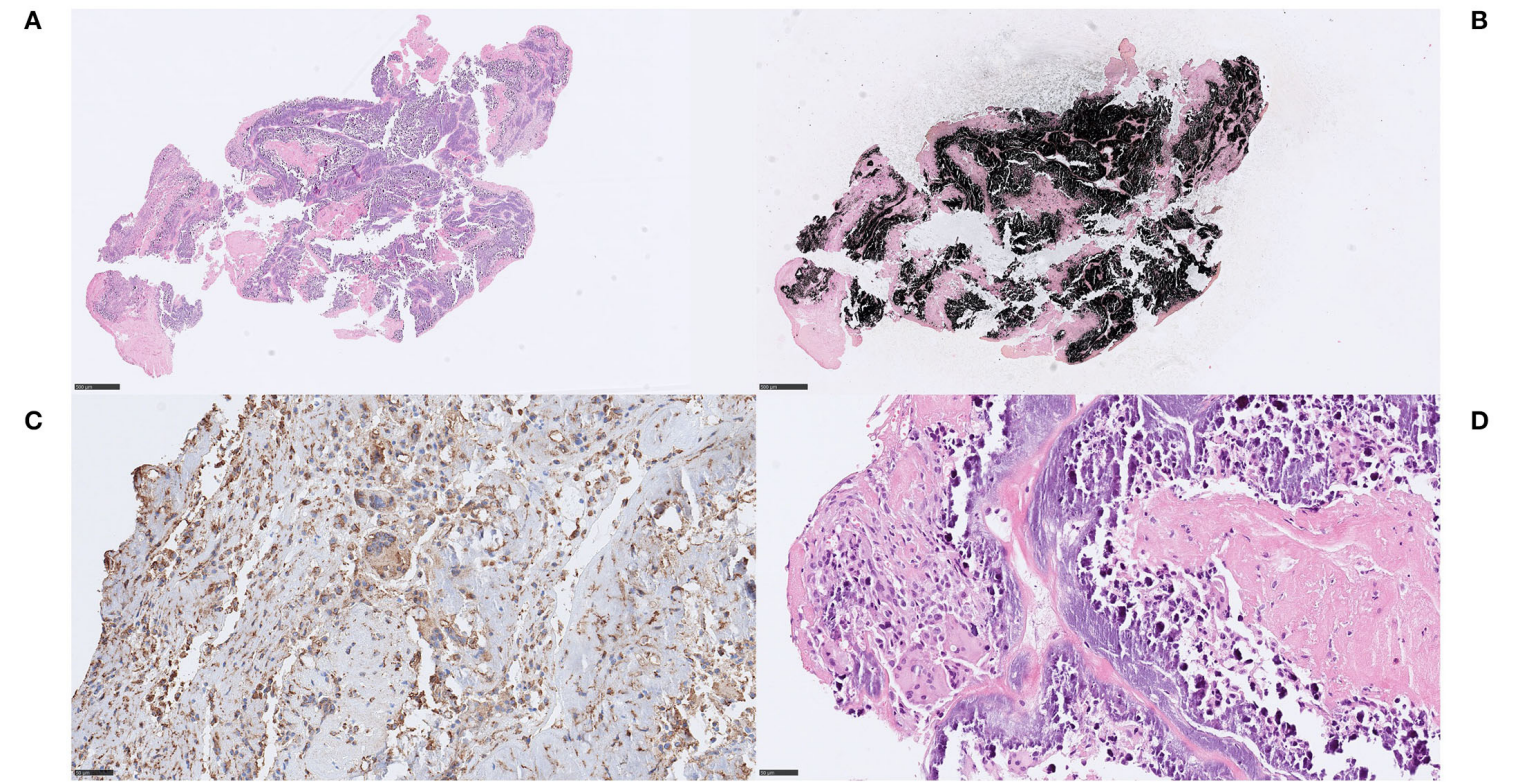
Extracellular DNA (ecDNA) with calcifications. Microcalcific type. Case 1.1: Microcalcific type displayed a dense network of ecDNA staining positive for the DNA stain Feulgen (not shown), intermixed with punctate, extracellular deposits of micromineralization **(A)** H&E, 50x; 3D, H&E, 400x. The micromineralizations associated with the ecDNA, as well as the ecDNA, stained heavily with von Kossa, confirming content of calcium **(B)**, von Kossa, 50x. Within the microcalcified component, a completely dominant cell population of anti-CD68-positive cells was evident **(C)**, CD68, 400x. These cells displayed an immunohistochemically estimated proliferation index of 50–60% with anti-Mib-1 (Ki67) (not shown). The findings, including the absence of neutrophils, indicate that this cell population consisted of macrophages exerting substantial ribosomal RNA synthesis, which in turn may suggest that macrophages could be the source of the ecDNA. Furthermore, frequent multi-nucleated giant cells were seen, however their pathophysiological role and significance in this setting is not certain. **(D)** H&E, 400x. *For 50x, scale bar* = *500* μ*m; for 400x, scale bar* = *50* μ*m*.

We noticed two types of calcified ecDNA-rich thrombi. These two types differed with respect to (1) general calcification morphology, (2) von Kossa staining pattern, (3) immunophenotype of macrophages and (4) presence of multinucleated giant cells (MNGCs). Based on these features, a large calcific type and a microcalcific type were distinguished. The large calcific type displayed large plaque-like calcifications and ecDNA that was lightly mineralized (Figures 1A–D). In addition, there was an abundance of myofibroblast-like cells with a CD45+/CD68+/α-SMA+ immunophenotype (Figures 2A–D) and absence of MNGCs. The microcalcific type showed punctate microcalcifications, heavily mineralized ecDNA and microcalcifications, a dominant cell population of CD45+/CD68+/α-SMA- macrophages and frequent MNGCs (Figures 3A–D). The macrophages were shown to have a significant proliferation index with anti-Mib-1 (Ki67) of 50–60%, indicating substantial ribosomal RNA synthesis (not shown). The ecDNA in all six cases stained consistently with Feulgen and both calcific types were H3Cit-immunonegative within the ecDNA.

#### Case 1.4–1.6

**Figure 4:**
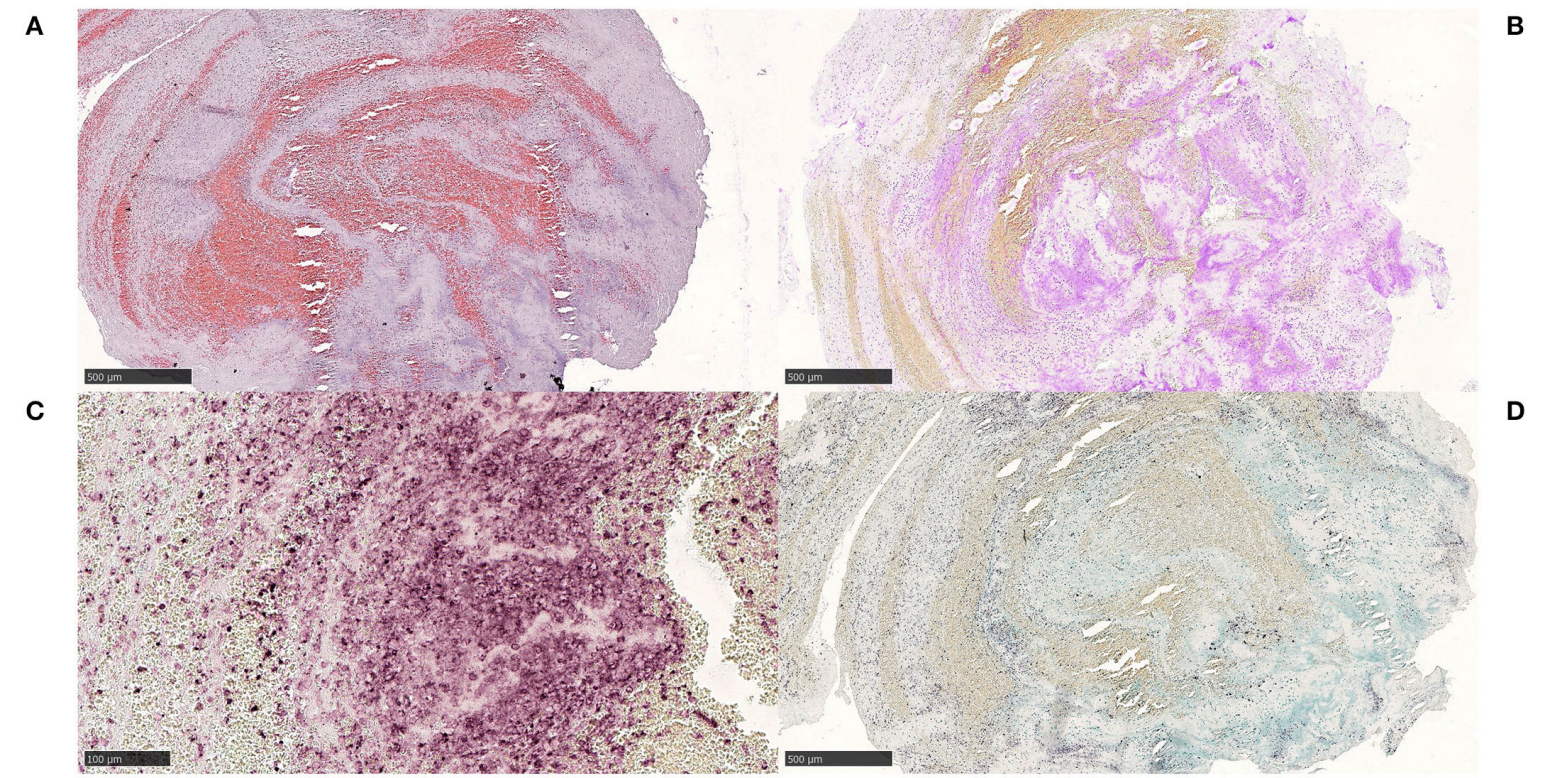
Extracellular DNA (ecDNA) without morphologically evident calcifications. Case 1.4 Webs of acellular basophilic material surrounding platelets and intermixed with red blood cells **(A)** H&E, 50x. A Feulgen stain confirmed the DNA content within this basophilic compartment, and it was regarded as ecDNA **(B)** Feulgen, 50x. Lymphocytic and neutrophilic cells in varying stages of degeneration, as well as shadows of necrotic cells were seen within the ecDNA in H&E (not shown). Immunohistochemically, the ecDNA, as well as the membranes of most viable cells, showed immunoreactivity for the pan-leukocytic marker CD45, indicating a leukocyte origin of the ecDNA **(C)** CD45, 200x. However, citrullinated H3 (H3Cit), a marker of neutrophil extracellular traps, did not stain the ecDNA, which suggests a non-neutrophil source **(D)** H3Cit, 50x. The stroke etiology and the underlying pathophysiological events are not evident. *For 50x, scale bar* = *500* μ*m; for 200x, scale bar* = *100* μ*m*.

In the non-mineralized ecDNA-rich cases, similar patterns of Feulgen-positive ecDNA were seen along with considerable numbers of degenerating lymphocytes near and within the ecDNA (Figures 4A–C). Within the ecDNA component, anti-H3Cit was negative in one of these cases (Figure 4D). No calcifications were identified.

##### Interpretation

The absence of H3Cit-immunoreactivity within ecDNA in four out of six cases suggests that cells other than neutrophils may be the source of extracellular nucleic acids in these cases. The von Kossa staining pattern suggests that the ecDNA is mineralized, possibly into hydroxyapatite (17, 18). The multitude of CD45+/CD68+/α-SMA+ cells in the large calcific type indicates a differentiation of monocytes/macrophages into myofibroblasts (19, 20). Both α-SMA- macrophages and α-SMA+ macrophages may contribute to the ecDNA in calcified thrombi. How these findings, and the findings in the non-mineralized ecDNA-rich thrombi, correlate to a history of atrial fibrillation and ongoing anticoagulant therapy is not clear.

### Cholesterol

#### Case 2.1–3

##### Clinical Data

Three cases with considerable amounts of cholesterol crystals were identified. All three patients were males with an average age of 75 years. Cardiovascular risk factors were present in two of the three patients, and these two also had a history of stroke/TIA. One patient had a history of atrial fibrillation but did not receive anticoagulation treatment for unclear reasons. One of the patients were treated with oral anti-platelets. At admission, these patients had occluded ICA-T, MCA, and BA, respectively, and the average NIHSS score was 22 (median 22). The patient with BA occlusion was intubated at admission. All underwent MT without prior rtPA. MT outcomes varied from a mTICI 0 to 3. Average number of passes were 3, 3 (median 2) and an average procedure time was calculated to 115 min (median time 110 min). 24 h post MT the average NIHSS was 21 and the patient with BA occlusion was then still sedated and intubated. Two of the patients died within 3 months after the MT, one patient due to SARS-CoV-2-related disease 4 weeks after the MT. The remaining patient was lost to clinical follow-up. Stroke etiology varied between large artery atherosclerosis, cryptogenic and other.

##### Histological Examination

**Figure 5:**
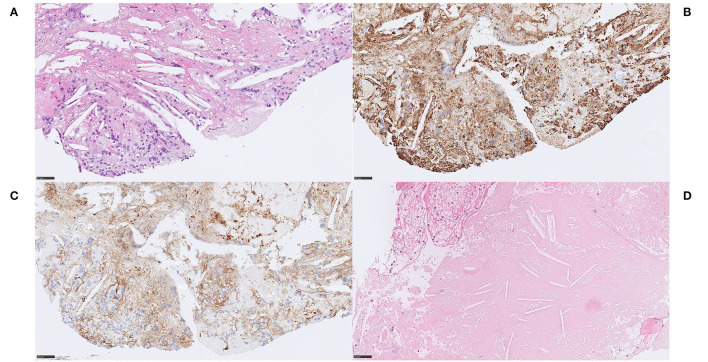
Cholesterol and necrosis. Case 2.3 Cholesterol crystals coalescing into lipid pools, surrounded by both fibrin and platelets **(A)** H&E, 400x. Cholesterol was accompanied by an inflammatory response, predominantly consisting of macrophages, as indicated immunohistochemically by anti-CD68 **(B)** CD68PG, 400x, but also lymphocytes and multinucleated giant cells that were identified in direct contact to some of the cholesterol clefts **(C)**, CD45, 400x. A few cells were immunoreactive for the endothelial marker endothelial transcription factor ETS-related gene (ERG), were noted in association to the inflammatory cells and to the cholesterol, possibly representing endothelial cells (not shown). Other components that may have been expected in embolized stroke thrombi material from an atherosclerotic plaque, such as fibrosis or evident parts of fibrous cap, were not identified. Composition of usual stroke elements was ordinary and unremarkable. Case 2.1 Multiple, large, and well-delineated areas of necrosis measuring up to 2 mm, containing cholesterol clefts within the necrotic debris **(D)**, H&E, 400x. Some mono-nuclear cells were neighboring the necrosis but there were no signs of an active or significant inflammatory response, indicating a short time from necrotic material entering the blood stream to thrombectomy. Red blood cells constituted 75% of the thrombus. Mineralization, common in atherosclerotic plaques, was not identified. *Scale bar* = *50* μ*m*.

In all three cases, large amounts of cholesterol were found both within necrotic debris and as coalesced cholesterol into lipid pools, as well as in direct contact with fibrin and platelets. In case 2.3, a foreign body reaction with some resemblance of a cholesterol granuloma was seen with cholesterol accompanied by an inflammatory response, predominantly by macrophages and to a lesser extent by lymphocytes and MNGCs (Figures 5A,B). The MNGCs were in direct contact with some of the cholesterol clefts, as visualized with anti-CD45 (Figure 5C). A few ERG-positive cells, possibly endothelial cells, were also noted in association to the inflammation and cholesterol (not shown). No fibrosis or evident parts of fibrous caps were identified. In case 2.2, cholesterol clefts were evident within large amounts of necrotic debris (Figure 5D). Overall, composition of the common stroke elements varied from 40 to 70%, the rest being cholesterol and necrosis.

##### Interpretation

Extracellular cholesterol form as crystalline aggregates and are washed out during routine tissue processing. This leaves behind biconvex needle-shaped empty clefts – a finding regarded as specific of cholesterol crystals. The presence of necrosis containing cholesterol clefts in stroke thrombi is indicative of an atherosclerotic plaque origin. A foreign body reaction is frequently found in atherosclerotic plaques, and MNGC's typically localize within regions of cholesterol, mineralization and necrotic debris. All three patients were hypertensive male patients. MT outcome varied but all had unfavorable clinical outcome; two of them died shortly after MT, due to a severe stroke in the posterior circulation and to SARS-CoV-2-related disease, respectively. Collectively, these findings were interpreted as emboli of atherosclerotic plaque contents, specifically of the necrotic lipid core.

### Adipocyte-Like Structures

#### Case 3.1–2

##### Clinical Data

Case 3.1 A 53-year-old male with a history of stroke and TIA, treated with oral anti-platelet medication, presented with signs of stroke and a NIHSS score of 5. Non-contrast CT and CT-angiography showed a left ICA-T occlusion without dense vessel sign. Intravenous thrombolysis was given prior to MT. A complete reperfusion (mTICI 3) was established within 108 min and four passes. NIHSS score at 24 h was 11 and the mRS at 90 days was assessed as 1. Stroke etiology according to TOAST was cryptogenic. Case 3.2 A 72-year-old female with a history of stroke/TIA, atrial fibrillation, diabetes, treated with oral anti-platelets. She presented to the hospital with signs of stroke and a NIHSS score of 19. CT-angiography showed an occlusion in the ICA and the patient was admitted to MT without pre-procedural intravenous thrombolysis. Complete reperfusion (mTICI 3) was established within 23 min and three passes using an aspiration device and a stent retriever in combination. NIHSS score at 24 h was three and the mRS at 90 days was 4. The etiology of the stroke according to TOAST was cryptogenic.

##### Histological Examination

**Figure 6:**
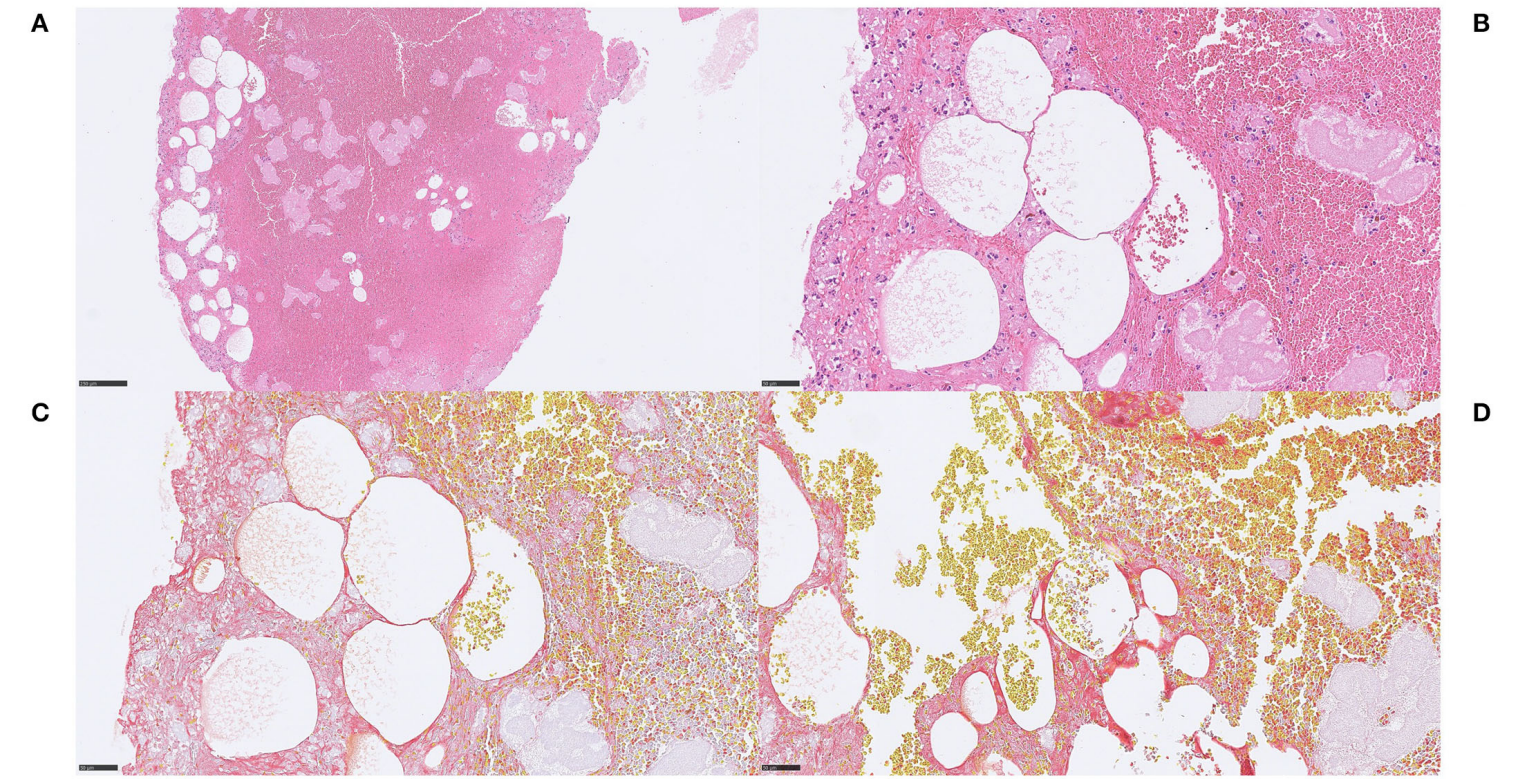
Adipocyte-like structures. Case 3.1 Aggregates of adipocyte-like structures within stroke thrombus **(A)**, H&E, 100x, some of these structures were juxtapositioned and separated by a very thin eosinophilic membrane-like structure **(B)**, H&E, 400x. The nature of this finding is not evident but no adipocyte nuclei were detected despite examination of multiple H&E-stained levels. Most adipocyte-like structures were scattered individually without a border structure toward surrounding red blood cells, and the membrane-like structures stained convincingly red on MSB, suggestive of fibrin content and an expansive process **(C,D)**, MSB, 400x. Immunohistochemical stains with S100 and Annexin were performed on multiple levels although no relevant immunoreactivity was obtained in membrane-like structures, as would have been expected if they were adipocytes (not shown). Some inflammatory cells, predominantly macrophages and some lymphocytes (immunohistochemically verified with CD45 and CD68; not shown) surrounded the spaces. No MNGCs were identified. These findings may suggest that the adipocyte-like structures may represent air emboli, which could have been introduced with repeated cerebral angiographies during the MT, either penetrating the occluding thrombus or incorporated in an occluding but still growing thrombus. Whether this in that case impacted the outcome of the MT is unclear. *For 100x, scale bar* = *250* μ*m; for 400x, scale bar* = *50* μ*m*.

On pathological examination, both thrombi had high contents of RBCs (85 and 60%, respectively). Aggregates of adipocyte-like structures were seen spread within the thrombi, each structure measuring 80–400 μm in diameter. Some of them juxtapositioned each other and were separated by only a very thin eosinophilic membrane-like structure (Figures 6A,B). No adipocyte nuclei were detected in relation to any of these spaces, and most of them were lying individually showing no bordering structure toward the surrounding red blood cells. Immunohistochemical stains with S100 and Annexin for adipocyte nuclei and membrane, respectively, were performed on multiple levels but no relevant immunoreactivity was obtained in these structures (not shown). However, the membrane-like structures stained homogenously red with MSB, indicative of fibrin content (Figures 6C,D). Inflammatory cells, predominantly macrophages and some lymphocytes, surrounded the adipocyte-like structures. Evident bone-marrow components or MNGCs were not identified.

##### Interpretation

These thrombi contained mainly rounded and sharply delineated empty spaces within areas of red blood cells, showing some resemblance of very large adipocytes/lipid globules on low power. However, we failed to prove the lipid origin of these spaces since evidence of adipocyte nuclei and/or cell membranes could not be established. Furthermore, MSB staining patterns indicated that the membrane-like structures were composed of fibrin. Nevertheless, these structures may represent washed out lipid material creating fat emboli, either as free lipid globules or as dying/dead adipocytes. Both these cases were RBC-rich thrombi and complete perfusion (mTICI 3) was achieved in both.

### Connective Tissue

#### Case 4.1

##### Clinical Data

A 73-year-old non-smoking male with a history of hypertension presented to the hospital with signs of stroke and a NIHSS score of 16. CT-angiography showed an occlusion of the right MCA M1 segment. Intravenous thrombolysis was administered prior to MT. A first pass recanalization (mTICI 3) was established in 8 min of procedure time. No device-related vessel damage was reported. NIHSS score at 24 h was 0 and the mRS at 90 days was 0. The etiology of the stroke according to the TOAST-criteria was cryptogenic.

##### Histological Examination

**Figure 7:**
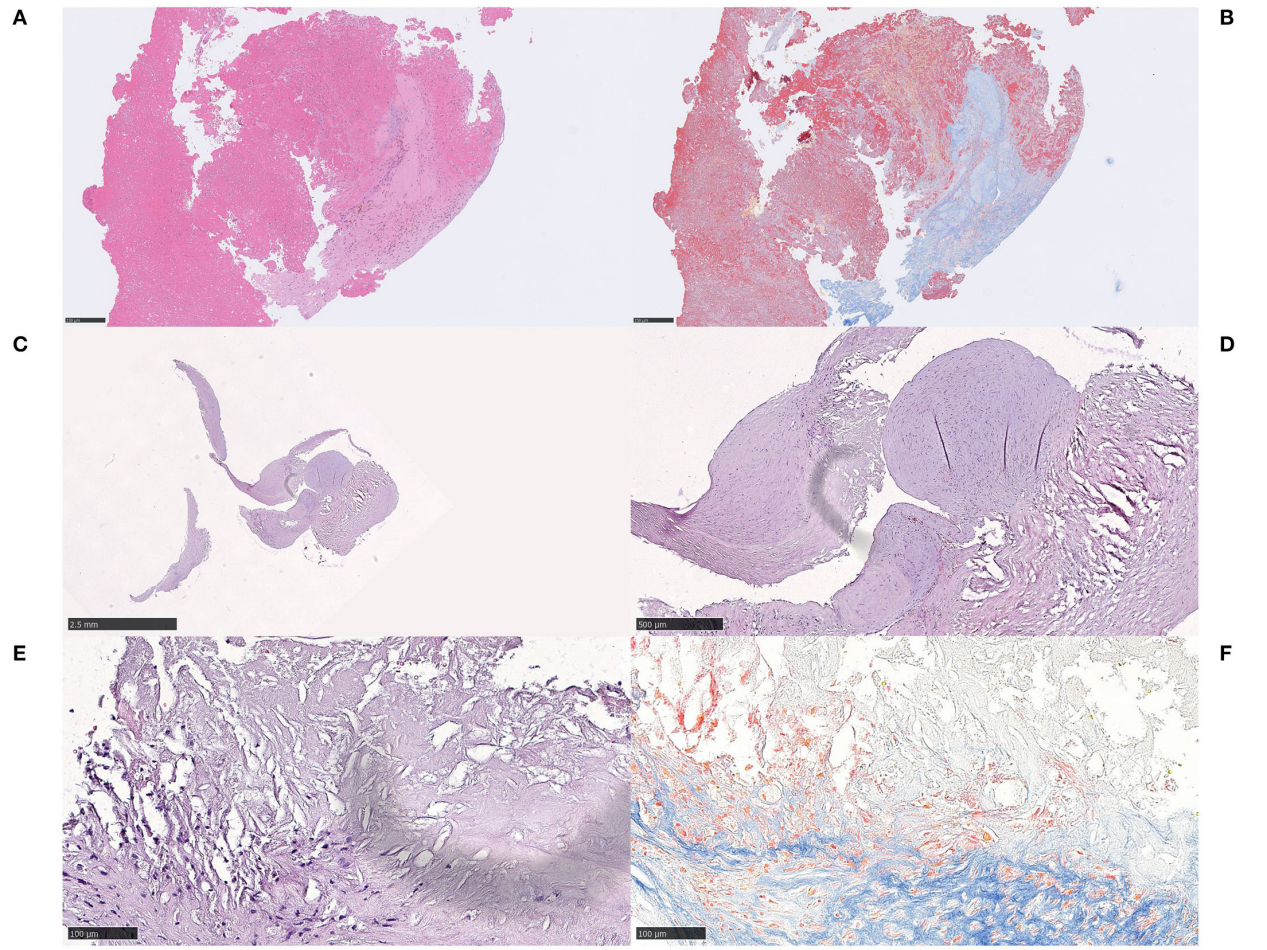
Connective tissue. Case 4.1 Fibrin-rich thrombus showing dense and light reticular fibrin patterns and fibrosis on H&E **(A)** H&E, 100x and MSB **(B)** MSB, 100x. On high power (not shown), the connective tissue was seen with spindled cells within an eosinophilic, collagenous, and focally myxoid background. A radial growth of dense connective tissue extended between strands of fibrin. In the border zone to the fibrin component, macrophages, hemosiderin-containing macrophages and lymphocytes were morphologically identified and immunohistochemically verified. Case 4.2 Curved-shaped fragments of connective tissue, measuring several millimeters in length **(C)** H&E, 12,5x. Focally, there was necrotic debris surrounded by the cap-shaped fibrotic tissue, containing lymphocytes, macrophages and spindled-shaped cells in the immediate proximity to the necrosis, as a fibrotic cap **(D)** H&E, 50x. On high power, the necrosis contained cholesterol-like clefts **(E,F)**, H&E and MSB, respectively, 200x. All connective tissue within the fibrotic compartment was composed of spindled cells within an eosinophilic, dense collagenous background with collagen staining convincingly blue on MSB (not shown). The findings in case 4.2 indisputably indicate embolized atherosclerotic content, specifically parts of the fibrous cap that once encaged the central core of lipids and necrotic debris of an atherosclerotic plaque. Red blood cells, fibrin and platelets made up about 5% of the thrombus material altogether. *For 12,5x, scale bar* = *2,5 mm; for 50x, scale bar* = *500* μ*m; for 100x, scale bar* = *250* μ*m; for 200x, scale bar* = *100* μ*m*.

A fibrin-rich thrombus (fibrin >70%) showing reticular patterns of fibrin of both dense and light qualities (Figure 7A). The thrombus also contained areas of connective tissue that stained blue on MSB, indicating collagen content, as well as structureless lightly eosinophilic material that stained light blue on MSB (Figure 7B). On high power, strands of collagen were seen to extend outwards from the connective tissue-core, surrounded by organized fibrin. The connective tissue was composed of spindled cells within an eosinophilic, collagenous, and focally myxoid background. In the border zone of the fibrin component, macrophages and hemosiderin-containing macrophages were identified, as well as lymphocytes (not shown). A hinted zonal pattern ranging from inflammation to quiescent dense fibrosis was noted, although no neutrophilic granulocytes could be identified.

#### Case 4.2

##### Clinical Data

A 52-year-old smoking male with no previous history of cerebrovascular disease presented to the hospital with signs of stroke and a NIHSS score of 22. CT-angiography showed a basilar artery occlusion. Intravenous thrombolysis was given prior to MT. Only minimal perfusion (mTICI 1) was established after three passes and 31 min of procedure time. No device-related vessel damage was reported. NIHSS at 24 h was scored as 1 and the mRS at 90 days as 1. The etiology of the stroke according to TOAST was cryptogenic.

##### Histological Examination

Histology of extracted material revealed curved-shaped fragments of connective tissue measuring several millimeters in length and 60–300 μm in thickness (Figure 7C). Focally, there was an increased number of lymphocytes, macrophages, and spindled cells in proximity to necrotic debris (Figure 7D). On high power, the necrosis appeared to contain cholesterol clefts (Figures 7E,F). The connective tissue was composed of spindled cells within an eosinophilic, dense collagenous background, staining blue on MSB, indicative of collagen content (not shown). Signs of small blood vessels were also noted within the fibrous material. Broadly attached to one of the curved-shaped connective tissue fragments, there was a rounded nodule containing spindled cells and scattered lymphocytes within a fibromyxoid background. Red blood cells, fibrin and platelets made up approximately 5% of the entire thrombus altogether.

##### Interpretation

In these two cases, we identified thrombi with significant amounts of connective tissue with an associated inflammatory response. Case 5.1 showed a very fibrin-rich clot with fibrotic organization. The amount and quality of fibrin, the number and distribution of inflammatory cells and the various stages of fibrosis indicate thrombus remodeling activity, i.e., an organized thrombus. The patient had a history of hypertension and presented with an M1-occlusion. The clot was successfully removed in 8 min (first pass) and the patient went from a NIHSS-score of 16 to 0 with excellent clinical outcome (mRS 0). We cannot rule out the possibility of this representing a part of a fibrous cap of an atherosclerotic plaque, parts of intimal thickening of a vascular wall or an embolus from the heart.

In case 5.2, we interpreted the findings as embolic content from an atherosclerotic plaque, specifically from the fibrous cap. The analyzed material also included the junction of the fibrous cap and parts of the necrotic lipid core. The nodule on top of the fibrous tissue was interpreted as an organized thrombus in a quiescent state, possibly representative of a previously healed plaque rupture.

### Myxomatous Material

#### Case 5

##### Clinical Data

A 79-year-old female with a history of hypertension and diabetes, treated with oral anti-platelet medication, presented to the hospital with symptoms of stroke, having a NIHSS score of 11. CT-angiography showed an occlusion of the left M2-segment of the MCA whereas a non-contrast CT revealed a dense vessel sign. The patient underwent MT without bridging intravenous thrombolysis. Five passes resulted in mTICI 2b recanalization after 190 min of procedure time. The NIHSS-score at 24 h post MT was 23. 2 weeks after the MT, the patient died of a cardiac arrest associated with an epileptic seizure. The etiology of the stroke according to TOAST was cryptogenic.

##### Histological Examination

**Figure 8:**
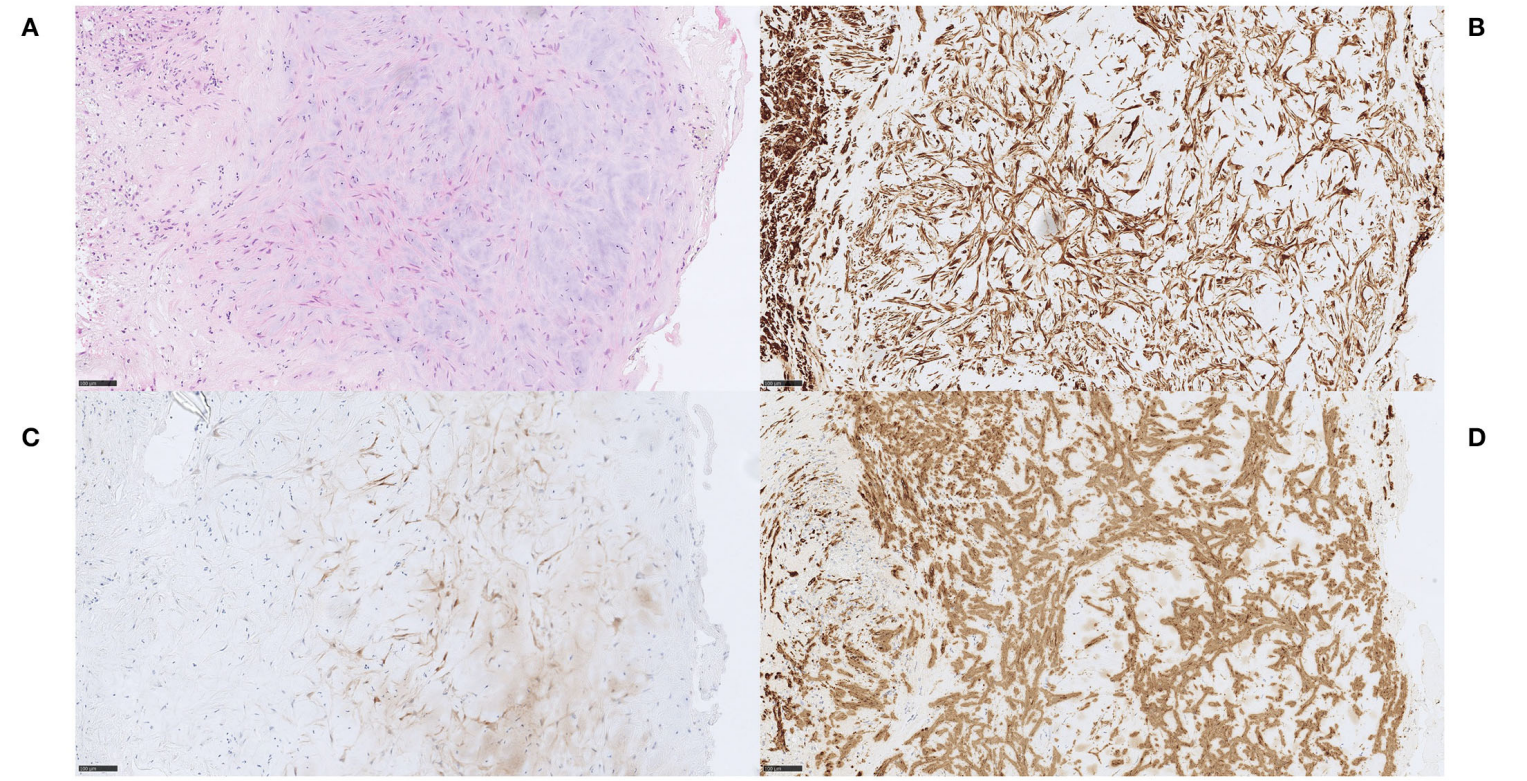
Myxomatous material. Case 5 Stellate cells with eosinophilic cytoplasm, arranged in cords and networks within a myxoid stroma, surrounded by aggregates of macrophages **(A)** H&E, 200x. Capillaries and scattered inflammatory cells were found within the myxoid stroma and glandular structures without mucin formation were identified focally. No mitotic activity, necrosis or hemosiderin deposits were seen. Stellate cells were consistently immunoreactive for Vimentin **(B)** Vimentin, 200x, PGP9.5 **(C)** PGP9.5, 200x and α-SMA **(D)** α-SMA, 200x, while variable or focal immunoreactivity for CD31 and calretinin were seen (not shown). NSE and S100 were negative (not shown). Vessels and capillaries showed immunoreactivity for CD31 and CD34 (not shown). The diagnosis of embolism of a cardiac myxoma in this case is not firmly established (no clinical data on cardiac tumor). We favored a cardiac myxoma embolism even though we cannot completely rule out the possibility of embolism of myxomatous material from other locations, e.g., cardiac valves and a carotid web. In our opinion, a definitive diagnosis of cerebral embolism of a cardiac myxoma should be made only after histopathological comparison between MT specimen and a surgically resected cardiac mass, confirming the primary diagnosis. *Scale bar* = *100* μ*m*.

Histology revealed a thrombus with a myxomatous area corresponding to half the thrombus size, with stellate cells arranged in cords and webs within the myxoid stroma (Figure 8A). These cells showed an abundant eosinophilic cytoplasm, round to oval nuclei with open chromatin and indistinct nucleoli. Capillaries and scattered inflammatory cells were found within the myxoid stroma. No mitotic figures or necrosis were identified. Aggregates of macrophages surrounded the myxomatous area. No hemosiderin deposits were seen. Focally, glandular structures were found, although not with mucin formation. Stellate cells showed immunoreactivity for Vimentin (Figure 8B), PGP9.5 (Figure 8C) and α-SMA (Figure 8D). Variable or focal immunoreactivity for CD31 and calretinin was noted (not shown). No immunoreactivity for NSE and S100 was found (not shown). Vessels and capillaries showed immunoreactivity for CD31 and CD34 (not shown).

##### Interpretation

Based on the histomorphology and immunohistochemical staining profile, we favored a cardiac myxoma to represent the myxomatous material in this case. However, due to limited material for histological analysis, we cannot rule out the possibility that this represents thromboembolism of material from cardiac valves with myxomatous degeneration, or an organizing thrombus. Furthermore, the cardiac status of the patient was not echocardiographically examined. Most importantly, there was no resected heart tumor specimen for histological comparison.

### Vascular Wall

#### Case 6

##### Clinical Data

A 52-year-old female without cardiovascular disease or cardiovascular risk factors, except for a patent foramen ovale, presented with an acute trauma-induced trimalleolar ancle fracture requiring orthopedic surgery the same day. 2 h post-surgery, she developed symptoms of stroke, with a NIHSS score of 8. CT-angiography showed an occlusion of the right MCA M2-segment, and a dense vessel sign was detected on non-contrast CT. The patient underwent MT with no previous intravenous thrombolysis, resulting in a near-complete reperfusion (mTICI 2c) in one pass and 40 min procedure time. No device-related vessel damage was reported. NIHSS-score at 24 h post MT was 3, and the mRS at 90 days was 0. Stroke etiology according to TOAST was classified as cryptogenic.

##### Histological Examination

**Figure 9:**
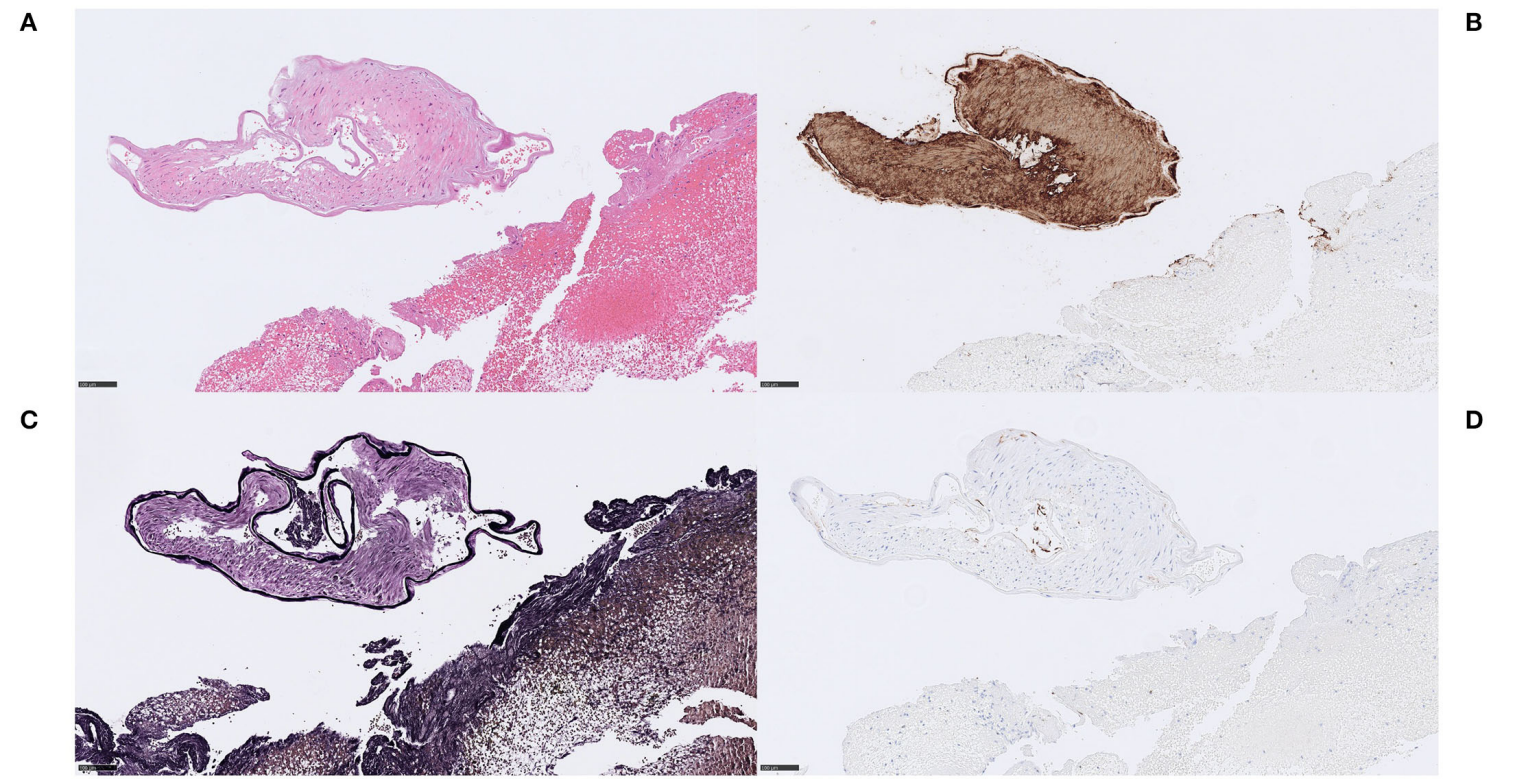
Vascular wall. Case 6 Isolated piece of tunica media with well-developed elastic lamina-like structure, no apparent pathological tunica intima and no evidence of tunica adventitia **(A)** H&E, 200x. The tunica media stained positive on the smooth muscle marker anti-α-SMA-immunostain, confirming smooth muscle cells **(B)** α-SMA, 200x. MSB staining indicated presence of thin collagen fibers between the smooth muscle cells (not shown). As suspected, Verhoeff van Gieson stained the morphologically identified internal elastic lamina in a dense, dark and linear fashion **(C)** Verhoeff van Gieson, 200x. A few endothelial cells could be seen resting on the elastic lamina in immunohistochemical stains with the endothelial marker CD34. These findings point to a conclusion of thrombectomized material of a healthy arterial wall, in this case most likely accidentally damaged during MT. **(D)** CD34, 200x. *Scale bar* = *100* μ*m*.

An RBC-rich thrombus (RBCs ~75%) containing a few isolated fragments consisting of smooth muscle cells that appeared organized with a maintained architecture resembling a tunica media. Toward the periphery of this fragment, ordinary tunica intima and internal elastic lamina-like structures were identified (Figures 9A,B). Staining with Verhoeff van Gieson confirmed the presence of an elastic lamina (Figure 9C). A few endothelial cells could be seen lining the surface in immunohistochemical stains with ERG and CD34 (Figure 9D). No tunica adventitia was identified, nor mineralized calcified material. There was no inflammation associated with the smooth muscle cells.

##### Interpretation

The findings of smooth muscle cells in a maintained tunica media-like architecture, an elastic lamina and an ordinary tunica intima with presence of endothelial cells, indicate parts of a healthy arterial wall, in this case most likely accidentally damaged during MT.

## Discussion

The purpose of this study was to present interesting and unusual histopathological observations in stroke thrombi from three European university hospital centers.

### ecDNA With and Without Calcifications

Substantial mineralization intermixed with considerable amounts of ecDNA occured rarely in all examined stroke thrombi (3/1,008). Generally, mineralization is a well-known phenomenon in the aortic valve, atherosclerotic plaques of large vessels, as well as in cerebral emboli. Histopathological analyses of calcifications in mechanically retrieved stroke thrombi are, however, scarce (7–11), and mineralization in addition to ecDNA has, to the best of our knowledge, only been described by Staessens et al. (11). Interestingly, all six cases that contained significant amounts of ecDNA (with or without mineralization) were elderly women with multiple cardiovascular risk factors, and five out of six were receiving anticoagulant therapy due to atrial fibrillation. This may be viewed as an interesting observation even though no conclusions on correlation can be drawn. The number of patients is very limited and anticoagulant medication is very common amongst ischemic stroke patients. It is, however, interesting to note that Heparin has been shown to dismantle the NET scaffold and prevent thrombus formation (21) but also to directly induce both lytic and vital NETs, depending on reactive oxygen species and neutrophil elastase activation (22). Whether direct factor Xa-inhibitors or vitamin K-antagonists impact the formation of extracellular traps remains unknown. Interestingly, all six patients presented with major stroke symptoms and despite technically fast and successful treatments, the clinical outcome in this group was mediocre (median mRS=3 at 90 days).

We identified two different types of mineralized, ecDNA-rich thrombi: a microcalcific and a large calcific type. Large numbers of macrophages accompanied mineralized ecDNA and calcifications in both types. An ordinary macrophage immunophenotype was displayed in the microcalcific type whereas an immunohistochemically proven macrophage-to-myofibroblast-transition was identified in the large calcific type. The implication of this is unknown, but it can hypothetically relate to the stroke etiology.

Activated platelets initiate a process that influences neutrophils to release extracellular traps, thereby contributing to the formation of thrombi in stroke patients (23). However, there are also other well-known sources of ecDNA, e.g., platelet mitochondria (24, 25), monocytes (26), as well as eosinophils, macrophages, mast cells and basophils (27). It is not possible to determine the precise source of the ecDNA in this study, but it can be concluded that; (I) not all ecDNA is derived from neutrophils, and (II) the ecDNA may represent a type of monocyte-extracellular traps (METs).

The relationship between cell populations, calcifications and ecDNA is not entirely clear. However, the immunophenotype of present cell population indicates that they are derived from circulating precursors rather than from SMCs within the atherosclerotic plaque, atrium of the heart or aortic valves, which would assumed to be immunonegative for the pan-leukocytic marker CD45. Formation of mineralization requires relatively long time and is most likely a result of pre-symptomatic processes within atherosclerotic plaque or aortic valves. Therefore, it seems plausible that plaque rupture in a blood vessel or a heart valve is the source of these emboli. Mineralized material exposed to the blood flow followed by intense colonization of circulating monocytes and an on-site rapid release of DNA as monocyte extracellular traps, may be the underlying chain of events. An alternative hypothesis is that the ecDNA is directly formed within a plaque or a cardiac valve. Such a thrombus may then also consist of other released intracellular components, e.g., calcium and actin filaments from various cell types undergoing necrosis.

### Cholesterol

The histopathological finding of cholesterol clefts strongly indicates embolization of necrotic lipid core contents from an atherosclerotic plaque. Foreign body reactions co-localize with mineralization and necrotic debris. Since cholesterol crystals triggers thrombosis (28), it may be speculated that the relative amount of cholesterol crystals may reflect the duration of exposed atherosclerotic elements after a plaque rupture. In one of the cases, material related to a necrotic lipid core constituted only 25% of the thrombus, the rest being the common components of stroke thrombi. This suggests a growing thrombus formation at the ruptured atherosclerotic plaque prior to embolization. Conversely, thrombi with relatively high content of cholesterol may indicate early embolization after plaque rupture.

### Adipocyte-Like Structures

Histologically, adipocyte-like structures are empty spaces of yet unknown nature. In our experience, they showed tendencies to occur within RBC-rich regions. The adipocyte-like structures were focally surrounded by macrophages, similarly to the crown-like structures previously described in adipose tissue (29). Fat embolism may be seen in orthopedic trauma patients but also after bone-marrow transplantation and liposuction, as well as in acute or chronic pancreatitis (30). None of our patients had a history of such interventions or disorders. The size of these structures (up to 400 μm in diameter) and the lack of evident adipocyte nuclei, questions the relationship to fat cells. Lack of evident cell membranes makes it unlikely that these are lipid globules of coalescing chylomicrons.

An alternative interpretation is that these empty spaces represent air bubbles. Such air emboli are difficult to prove from a histopathological point of view but microscopic, asymptomatic (average diameter 30–60 μm) as well as larger and symptomatic air embolism has been reported in conjunction with cerebral angiography (31–33). This may suggest that the repeated cerebral angiographies resulted in small amounts of air emboli during the MT that either penetrated the occluding thrombus or were incorporated in an occluding but still growing thrombus. Whether this in that case impacted the outcome of the MT is unclear.

### Connective Tissue

Presence of connective tissue may indicate remodeling activity in an organizing thrombus. Alternative interpretations include pathologic intimal thickening from an *in-situ* occlusion or embolism of a fibrous cap from an atherosclerotic plaque or a carotid web (34). The two patients were both males with hypertension and smoking as the only known cardiovascular risk factors, in which the ischemic strokes were considered clinically as cryptogenic. In contrast, the histopathological findings indisputably indicated embolized atherosclerotic content in one of the cases, speaking in favor of histopathological examinations in some cases may help to identify the source of the ischemic event.

### Myxomatous Material

Identifying stroke thrombi containing myxomatous material may be important since they may emanate from a cardiac myxoma, and if so, diagnostic work-up, treatment and general patient outlook differ from most other stroke patients. Histopathological studies of embolized cardiac myxoma are infrequent (9, 35, 36), and the morphology and immunohistochemical profile of the specimens differs in these studies. In our case presented in this study, the histomorphology and immunohistochemical staining pattern favored a cardiac myxoma embolism even though we cannot completely rule out the possibility of myxomatous embolism from other locations, e.g., cardiac valves and a carotid web. In our opinion, a definitive diagnosis of cerebral embolism of a cardiac myxoma should be made only after histopathological comparison between MT specimen and a surgically resected cardiac mass, confirming the primary diagnosis.

### Vascular Wall

Although there were theoretical conditions of an embolization of venous wall components in case 6, the histopathological examination indicated vascular wall components of a small, muscular artery, most likely related to the mechanical thrombectomy procedure. Presence of vascular wall components in stroke thrombi described as “banded collagen fibers with distinct boundary existing at the margin or outside of the thrombus,” has been described by Funatsu et al. as a quite common histological finding in retrieved stroke thrombi (14). Variations in the definition of vascular wall structures may explain different study results but the presence of such structures may also depend on MT devices and technique. Whether vessel wall embolization or *in-situ* inclusion of vessel wall components into the thrombus is a common phenomenon in MT remains unclear. This patient suffered from ischemic stroke soon after orthopedic surgery but as the incidence of pulmonary embolism after such operations are low (37) there may be no causality in this case but a mere coincidence.

Finally, two out of 15 patients included in this study died of SARS-CoV-2 virus during the COVID-19-pandemic. Associations between SARS-CoV-2 and large artery occlusion are yet to be found. The retrospective study design of this case series does not allow us to draw any conclusions on whether SARS-CoV-2 virus impacts on the risk of large artery occlusion or whether it affects the composition of the stroke thrombi.

### Limitations

The findings in this study should be interpreted in the context of its retrospective nature and study design. In addition, there are some obvious limitations of this study. Firstly, irretrievable thrombi are not assessed histopathologically. Secondly, variations in formalin fixation, handling and staining protocols of the specimens may lead to variations in morphology and immunoreactivity, which in turn may influence the histopathological assessment. Furthermore, the clinical and radiological data and stroke etiology were self-reported at each center which is a source of variability.

Strengths of this study are the multi-center design and the considerable number of thrombi that has been reviewed.

## Conclusion

Retrospective histopathological characterization of stroke thrombi revealed unusual features that related to distinct cardiovascular diseases, patient characteristics, and MT technique. Specifically, we identified two different types of mineralized, ecDNA-rich thrombi, accompanied by differentially differentiated macrophages, in elderly women with atrial fibrillation receiving anticoagulant therapy. More knowledge about these components may increase our understanding of stroke pathophysiology and influence interventional technique.

## Data Availability Statement

The data analyzed in this study is subject to the following licenses/restrictions: The clinical stroke thrombi material belongs to the hospitals. Requests to access these datasets should be directed to OA, oskar.aspegren@regionstockholm.se.

## Ethics Statement

The studies involving human participants were reviewed and approved by Swedish Research Ethics Board (2020-03349) AZGH Ethical Committee (AZGS2015065) French Ethical Committee (2019-A00414-53). Written informed consent for participation was not required for this study in accordance with the national legislation and the institutional requirements.

## Author Contributions

OA performed histological examination of all stroke thrombi and wrote the manuscript. Cases from Lille and AZ Groeninge are discussed between OA, SS, and SV. TA, FA, SS, and SV have reviewed the manuscript and made comments and improvements. LD has been responsible for additional laboratory work at Kulak. All authors contributed to the article and approved the submitted version.

## Conflict of Interest

The authors declare that the research was conducted in the absence of any commercial or financial relationships that could be construed as a potential conflict of interest.

## Publisher's Note

All claims expressed in this article are solely those of the authors and do not necessarily represent those of their affiliated organizations, or those of the publisher, the editors and the reviewers. Any product that may be evaluated in this article, or claim that may be made by its manufacturer, is not guaranteed or endorsed by the publisher.

## Abbreviations

BA: Basilar artery
ecDNA: Extracellular deoxyribonucleic acid
ICA: Internal carotid artery
ICA-T, Internal carotid artery: T-occlusion
MCA: Middle cerebral artery
MNGCs: Multinucleated giant cells
MT: Mechanical thrombectomy
NETs: Neutrophil extracellular traps
rtPA: Recombinant tissue plasminogen activator.

## References

[1] Berkhemer OA Fransen PS Beumer D van den Berg LA Lingsma HF Yoo AJ. MR clean investigators. a randomized trial of intraarterial treatment for acute ischemic stroke. N Engl J Med. (2015) 372:11–20. 10.1056/NEJMoa1411587 25517348

[2] Campbell BC Mitchell PJ Kleinig TJ Dewey HM Churilov L Yassi N. ExtenD-IA investigators. Endovascular therapy for ischemic stroke with perfusion-imaging selection. N Engl J Med. (2015) 372:1009–18. 10.1056/NEJMoa1414792 25671797

[3] Goyal M Demchuk AM Menon BK Eesa M Rempel JL Thornton J. Escape trial investigators. Randomized assessment of rapid endovascular treatment of ischemic stroke. N Engl J Med. (2015) 372:1019–30. 10.1056/NEJMoa1414905 25671798

[4] Jovin TG Chamorro A Cobo E de Miquel MA Molina CA Rovira A. Revascat trial investigators. Thrombectomy within 8 h after symptom onset in ischemic stroke. N Engl J Med. (2015) 372:2296–306. 10.1056/NEJMoa1503780 25882510

[5] Saver JL Goyal M Bonafe A Diener HC Levy EI Pereira VM. Swift prime investigators. stent-retriever thrombectomy after intravenous t-PA vs. t-PA alone in stroke. N Engl J Med. (2015) 372:2285–95. 10.1056/NEJMoa1415061 25882376

[6] Staessens S Fitzgerald S Andersson T Clarençon F Denorme F Gounis MJ. Histological stroke clot analysis after thrombectomy: technical aspects and recommendations. Int J Stroke. (2020) 15:467–76. 10.1177/1747493019884527 31679478

[7] Almekhlafi MA Hu WY Hill MD Auer RN. Calcification and endothelialization of thrombi in acute stroke. Ann Neurol. (2008) 64:344–8. 10.1002/ana.21404 PMID: 18570298 18570298

[8] Mereuta OM Rossi R Douglas A Gil SM Fitzgerald S Pandit A. Characterization of the 'white' appearing clots that cause acute ischemic stroke. J Stroke Cerebrovasc Dis. (2021) 30:106127. 10.1016/j.jstrokecerebrovasdis.2021.106127 34592611

[9] Mak G Lu JQ Perera K. Histopathologic analysis of retrieved cerebral thrombi in acute ischemic stroke patients with proximal anterior circulation occlusions amenable to endovascular thrombectomy. J Neurol Sci. (2021) 429:117617. 10.1016/j.jns.2021.117617 34461551

[10] Genchi A Schwarz G Semerano A Callea M Sanvito F Simionato F. Large vessel occlusion stroke due to dislodged aortic valve calcification revealed by imaging and histopathology. J Neurol Sci. (2020) 408:116573. 10.1016/j.jns.2019.116573 31731112

[11] Staessens S François O Desender L Vanacker P Dewaele T Sciot R. Detailed histological analysis of a thrombectomy-resistant ischemic stroke thrombus: a case report. Thromb J. (2021) 19:11. 10.1186/s12959-021-00262-1 33618719 PMC7901204

[12] Staessens S Denorme F Francois O Desender L Dewaele T Vanacker P. Structural analysis of ischemic stroke thrombi: histological indications for therapy resistance. Haematologica. (2020) 105:498–507. 10.3324/haematol.2019.219881 31048352 PMC7012484

[13] Di Meglio L Desilles JP Solonomenjanahary M Labreuche J Ollivier V Dupont S. DNA content in ischemic stroke thrombi can help identify cardioembolic strokes among strokes of undetermined cause. Stroke. (2020) 51:2810–6. 10.1161/STROKEAHA.120.029134 32811390

[14] Funatsu N Hayakawa M Hashimoto T Yamagami H Satow T Takahashi JC. Vascular wall components in thrombi obtained by acute stroke thrombectomy: clinical significance and related factors. J Neurointerv Surg. (2019) 11:232–6. 10.1136/neurintsurg-2018-014041 30097483

[15] Wilson JT Hareendran A Grant M Baird T Schulz UG Muir KW. Improving the assessment of outcomes in stroke: use of a structured interview to assign grades on the modified Rankin Scale. Stroke. (2002) 33:2243–6. 10.1161/01.str.0000027437.22450.bd 12215594

[16] Adams HP Jr Bendixen BH Kappelle LJ Biller J Love BB Gordon DL Marsh EE 3rd. Classification of subtype of acute ischemic stroke. Definitions for use in a multicenter clinical trial. TOAST. Trial of org 10,172 in acute stroke treatment. Stroke. (1993) 24:35–41. 10.1161/01.str.24.1.35 7678184

[17] Bertran O del Valle LJ Revilla-López G Chaves G Cardús L Casas MT. Mineralization of DNA into nanoparticles of hydroxyapatite. Dalton Trans. (2014) 43:317–27. 10.1039/c3dt52112e 24105025

[18] Coscas R Bensussan M Jacob MP Louedec L Massy Z Sadoine J. Free DNA precipitates calcium phosphate apatite crystals in the arterial wall *in vivo*. Atherosclerosis. (2017) 259:60–7. 10.1016/j.atherosclerosis.2017.03.005 28292668

[19] Vierhout M Ayoub A Naiel S Yazdanshenas P Revill SD Reihani A. Monocyte and macrophage derived myofibroblasts: is it fate? A review of the current evidence. Wound Repair Regen. (2021) 29:548–62. 10.1111/wrr.12946 34107123

[20] Meng XM Wang S Huang XR Yang C Xiao J Zhang Y. Inflammatory macrophages can transdifferentiate into myofibroblasts during renal fibrosis. Cell Death Dis. (2016) 7:e2495. 10.1038/cddis.2016.402 27906172 PMC5261004

[21] Fuchs TA Brill A Duerschmied D Schatzberg D Monestier M Myers DD Jr. Extracellular DNA traps promote thrombosis. Proc Natl Acad Sci USA. (2010) 107:15880–5. 10.1073/pnas.1005743107 20798043 PMC2936604

[22] Lelliott PM Momota M Shibahara T Lee MSJ Smith NI Ishii KJ Coban C. Heparin induces neutrophil elastase-dependent vital and lytic NET formation. Int Immunol. (2020) 32:359–68. 10.1093/intimm/dxz084 31879779

[23] Nakazawa D Desai J Steiger S Müller S Devarapu SK Mulay SR. Activated platelets induce MLKL-driven neutrophil necroptosis and release of neutrophil extracellular traps in venous thrombosis. Cell Death Discov. (2018) 4:6. 10.1038/s41420-018-0073-2 30062055 PMC6060161

[24] Boudreau LH Duchez AC Cloutier N Soulet D Martin N Bollinger Jet al. Platelets release mitochondria serving as substrate for bactericidal group IIA-secreted phospholipase A2 to promote inflammation. Blood. (2014) 124:2173–83. 10.1182/blood-2014-05-573543 25082876 PMC4260364

[25] McArthur K Whitehead LW Heddleston JM Li L Padman BS Oorschot V. BAK/BAX macropores facilitate mitochondrial herniation and mtDNA efflux during apoptosis. Science. (2018) 359:eaao6047. 10.1126/science.aao6047 29472455

[26] Granger V Faille D Marani V Noël B Gallais Y Szely N. Human blood monocytes are able to form extracellular traps. J Leukoc Biol. (2017) 102:775–81. 10.1189/jlb.3MA0916-411R 28465447

[27] Ramos-Martínez E Hernández-González L Ramos-Martínez I Pérez-Campos Mayoral L López-Cortés GI Pérez-Campos E. Multiple origins of extracellular DNA traps. Front Immunol. (2021) 12:621311. 10.3389/fimmu.2021.621311 33717121 PMC7943724

[28] Shi C Kim T Steiger S Mulay SR Klinkhammer BM Bäuerle T. Crystal clots as therapeutic target in cholesterol crystal embolism. Circ Res. (2020) 126:e37–52. 10.1161/CIRCRESAHA.119.315625 32089086

[29] Wang L Zhao RP Song XY Wu WF. Targeting ERβ in Macrophage Reduces crown-like structures in adipose tissue by inhibiting osteopontin and HIF-1α. Sci Rep. (2019) 9:15762. 10.1038/s41598-019-52265-8 31673032 PMC6823357

[30] Adeyinka A Pierre L. Fat Embolism. 2021 Nov 3. In: StatPearls [Internet]. Treasure Island, Florida, FL: StatPearls Publishing (2022).

[31] Markus H Loh A Israel D Buckenham T Clifton A Brown MM. Microscopic air embolism during cerebral angiography and strategies for its avoidance. Lancet. (1993) 341:784–7. 10.1016/0140-6736(93)90561-t 8096000

[32] Gupta R Vora N Thomas A Crammond D Roth R Jovin T. Symptomatic cerebral air embolism during neuro-angiographic procedures: incidence and problem avoidance. Neurocrit Care. (2007) 7:241–6. 10.1007/s12028-007-0041-9 17805494

[33] Segan L Permezel F. Ch'ng W, Millar I, Brooks M, Lee-Archer M, Cloud G. Cerebral arterial gas embolism from attempted mechanical thrombectomy: recovery following hyperbaric oxygen therapy. Pract Neurol. (2018) 18:134–6. 10.1136/practneurol-2017-001828 29288212

[34] Semerano A Mamadou Z Desilles JP Sabben C Bacigaluppi M Piotin M. Carotid webs in large vessel occlusion stroke: clinical, radiological, and thrombus histopathological findings. J Neurol Sci. (2021) 427:117550. 10.1016/j.jns.2021.117550 34175777

[35] Garcia-Ptacek S Matias-Guiu JA Valencia-Sánchez C Gil A Bernal-Becerra I De las Heras-Revilla V. Mechanical endovascular treatment of acute stroke due to cardiac myxoma. J Neurointerv Surg. (2014) 6:e1. 10.1136/neurintsurg-2012-010343 22791184

[36] Baek SH Park S Lee NJ Kang Y Cho KH. Effective mechanical thrombectomy in a patient with hyperacute ischemic stroke associated with cardiac myxoma. J Stroke Cerebrovasc Dis. (2014) 23:e417–9. 10.1016/j.jstrokecerebrovasdis.2014.05.006 25174564

[37] Zaidi R MacGregor A Cro S Goldberg A. Pulmonary embolism and mortality following total ankle replacement: a data linkage study using the NJR data set. BMJ Open. (2016) 6:e011947. 10.1136/bmjopen-2016-011947 27329444 PMC4916628

